# The Role of Autophagy in the Pathogenesis of Diabetic Nephropathy

**DOI:** 10.1155/2013/193757

**Published:** 2013-12-17

**Authors:** Kosuke Yamahara, Mako Yasuda, Shinji Kume, Daisuke Koya, Hiroshi Maegawa, Takashi Uzu

**Affiliations:** ^1^Department of Medicine, Shiga University of Medical Science, Tsukinowa-Cho, Seta, Otsu, Shiga 520-2192, Japan; ^2^Diabetes & Endocrinology, Kanazawa Medical University, Daigaku 1-1, Kahoku-Gun, Uchinada, Ishikawa 920-0293, Japan

## Abstract

Diabetic nephropathy is a leading cause of end-stage renal disease worldwide. The multipronged drug approach targeting blood pressure and serum levels of glucose, insulin, and lipids fails to fully prevent the onset and progression of diabetic nephropathy. Therefore, a new therapeutic target to combat diabetic nephropathy is required. Autophagy is a catabolic process that degrades damaged proteins and organelles in mammalian cells and plays a critical role in maintaining cellular homeostasis. The accumulation of proteins and organelles damaged by hyperglycemia and other diabetes-related metabolic changes is highly associated with the development of diabetic nephropathy. Recent studies have suggested that autophagy activity is altered in both podocytes and proximal tubular cells under diabetic conditions. Autophagy activity is regulated by both nutrient state and intracellular stresses. Under diabetic conditions, an altered nutritional state due to nutrient excess may interfere with the autophagic response stimulated by intracellular stresses, leading to exacerbation of organelle dysfunction and diabetic nephropathy. In this review, we discuss new findings showing the relationships between autophagy and diabetic nephropathy and suggest the therapeutic potential of autophagy in diabetic nephropathy.

## 1. Introduction

The increasing prevalence of diabetes mellitus and its vascular complications has become a major health problem worldwide. Diabetic nephropathy is a serious complication of diabetes and is a common cause of end-stage renal disease. Diabetes induces glomerular damage, along with proteinuria, and subsequent tubulointerstitial lesions, leading to end-stage renal disease [1–3]. Initially, the patient shows hyperfiltration, represented by high glomerular filtration rates (GFRs) and occasional occurrence of microalbuminuria. Later, the patient shows a gradual decline in the GFR and persistence of microalbuminuria that comes before mild and subsequently moderate proteinuria. Urinary protein seems to be almost entirely reabsorbed in early and late proximal tubules and may induce tubulointerstitial damage [3]. Reducing proteinuria by keeping blood pressure and blood glucose levels under control is therefore a primary therapeutic goal with diabetic nephropathy [4, 5]. Unfortunately, however, some patients develop treatment-resistant proteinuria, resulting in end-stage renal disease. There is now an urgent need to identify new therapeutic target molecules or cellular processes that underlie the pathogenesis of diabetic nephropathy to establish additional therapeutic options.

Autophagy has recently been found to be a stress-responsive intracellular system, because it is likely that the disturbance of this machinery is involved in the pathogenesis of age- and diabetes-related diseases [6, 7]. Autophagy is a part of the catabolic processes that degrades damaged intracellular proteins and organelles [8]. Accumulating evidence suggests that autophagy activity declines in some organs under obesity conditions, and the functional roles of autophagy in the kidney have been gradually clarified. It has been reported that autophagy has a protective function against renal damage induced by aging [9, 10], hypoxia [11, 12], and anticancer drugs [13–15]. However, the relationship between autophagy and diabetic nephropathy remains to be elucidated, although several recent papers have suggested that autophagy machinery is involved in the pathogenesis of diabetic nephropathy. In this review, we summarize and discuss recent findings on the role of autophagy in diabetic nephropathy.

## 2. Autophagy

The term “autophagy” is derived from Greek and means self-eating. Autophagy is highly conserved from yeast to mammals. It is a bulk degradation process involved in the clearance of damaged proteins and organelles. Autophagy works to maintain cell homeostasis under various stress conditions. Three types of autophagy have been identified in cells: macroautophagy, microautophagy, and chaperone-mediated autophagy. All types differ in their mechanisms and functions [16, 17]. Of the three types, macroautophagy is the most prevalent and in this review is referred to as autophagy.

**Figure 1:**
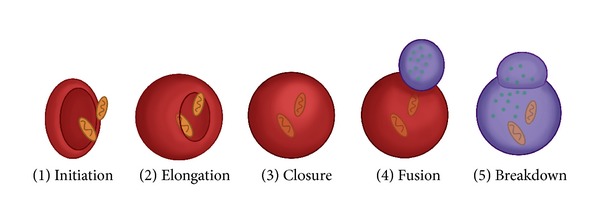
Steps of the autophagic pathways. Five steps in the autophagic pathways have been identified: (1) initiation, isolated membrane appears in cytosol; (2) elongation, elongation is characterized by membrane bending and an increase in the size of the phagophore; (3) closure, the autophagosome membrane wraps around the cytosolic components; (4) fusion, the fusion of the autophagosome with a lysosome to form an autolysosome; and (5) breakdown, the autolysosome is degradated by lysosomal hydrolases.

**Figure 2:**
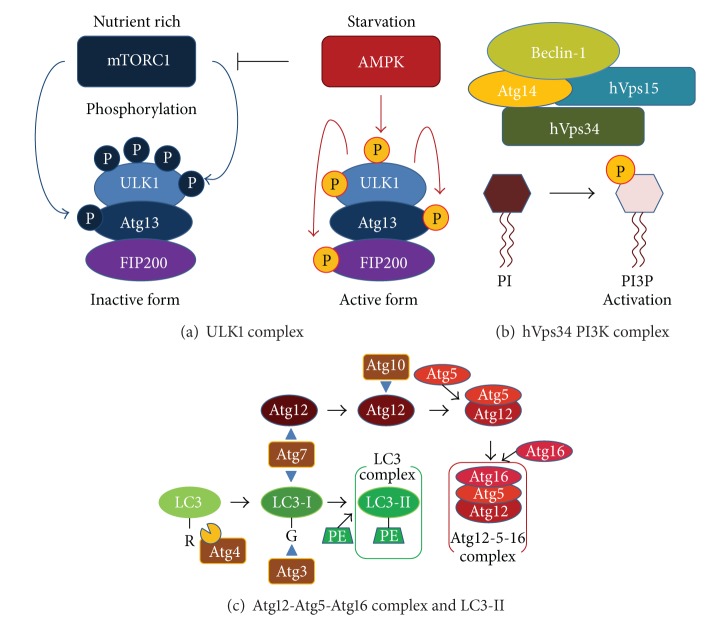
Autophagy regulation. (a) ULK1 protein kinase complex. ULK1 is a critical regulator of nutrient-related autophagy. mTOR-dependent phosphorylation of ULK1 (Atg1) and Atg13 under nutrient-rich conditions inhibits autophagy. In contrast, AMPK-dependent phosphorylation of ULK1 activates autophagy induction under energy-depleted condition. (b) PI3K complex. Phosphatidylinositol 3-kinase (PI3K) phosphorylates phosphatidylinositol in the membrane lipid to create phosphatidylinositol 3-phosphate. Class III PI3K comprises hVps34, hVps15, Beclin-1, and Atg14. (c) Atg12-Atg5-Atg16 complex and LC3-II. Unlike other ubiquitin-like proteins, the ubiquitin-like protein Atg12 has a C-terminal glycine, which protects it from processing. Atg12 is conjugated to the substrate Atg5 by Atg7 and Atg10. The Atg12-Atg5 conjugate forms a complex with Atg16. Self-oligomerization of Atg16 results in a multimer of the Atg12-Atg5-Atg16 complex. After the ubiquitin-like protein LC3 has had its C-terminal arginine residue cleaved by the cysteine protease Atg4, it is passed on to Atg7 and Atg3 and transferred into the head group of its substrate phosphatidylethanolamine (PE). This LC3-PE conjugate functions as part of the membrane component of the autophagosome. When LC3-PE is once again deconjugated to PE by Atg4, Atg8 is recycled.

During autophagy, *de novo* isolation membranes (phagophores) elongate and fuse while engulfing a portion of the cytoplasm within double-membrane vesicles (autophagosomes). The origin of the autophagosomal membrane is likely to be the endoplasmic reticulum (ER) membrane [18]. Five major steps are involved in the formation of autophagosomes: initiation, elongation, closure, fusion, and breakdown (Figure 1). During these steps, autophagy-related genes (Atg) and proteins are involved. Autophagy is initiated by the unc-51-like kinase (Ulk) 1 (the mammalian ortholog of yeast Atg1) complex, which comprises Ulk1 Ser/Thr protein kinase, Atg13, and FIP200 (mammalian homolog of yeast Atg17) (Figure 2(a)) [19–21]. Phosphorylation of Atg13 and FIP200 by Ulk1 is essential to trigger autophagy. Phagophore nucleation is dependent on Beclin 1 (Atg6 in yeast), an hVps34 or class III phosphatidylinositol 3-kinase (PI3 K) complex, which comprises hVps34, hVps15, Beclin 1, and Atg14 (Figure 2(b)) [22, 23]. During autophagosome elongation/closure, two dependent ubiquitin-like conjugation systems are involved: Atg12 and LC3 (the mammalian ortholog of yeast Atg8) [24].

The Atg12-Atg5 conjugate, which forms the Atg12-Atg5-Atg16 complex, contributes to the stimulation and localization of the LC3 conjugation reaction. The cytosolic isoform of LC3 (LC3-I) is conjugated to phosphatidylethanolamine through two consecutive ubiquitin-like reactions catalyzed by E1-like enzyme Atg7 and the E2-like enzyme Atg3 to form LC3-II (Figure 2(c)) [25]. Thus, LC3-II formation is recognized as a marker of the existence of autophagosomes in cell or animal experiments [26–28]. After formation, autophagosomes merge with the lysosomal compartment to form autolysosomes. The protein p62, also known as sequestosome 1, is known to localize to autophagosomes via LC3 interaction and to be constantly degraded by the autophagy-lysosome system [29, 30]. The accumulation of p62 is observed in autophagy-deficient cells [29, 30].

## 3. Mechanisms of Autophagy Regulation

Autophagy is upregulated in response to nutrient starvation and extracellular or intracellular stress. In this section, we outline the regulatory mechanism underlying nutrient starvation- and stress-induced autophagy activation in cells.

During nutrient deprivation, autophagosome formation is dramatically induced. In both yeast and mammalian cells, two well-characterized signaling cascades that sense nutrient status, the mammalian target of rapamycin (mTOR) complex 1 (mTORC1) and AMP activated protein kinase (AMPK) pathways, are potent regulators of autophagy. Autophagy is induced by AMPK, which is a key energy sensor of AMP, and is upregulated by an increase in intracellular levels of AMP [31]. Conversely, autophagy is inhibited by mTORC1, a central cell growth regulator that integrates growth factor and hypernutrient signals [32–34].

AMPK monitors the energy condition of a cell by sensing the AMP/ATP ratio [35]. Autophagy is activated with low-glucose conditions in cultured cells [36]. Under glucose deprivation, ATP concentrations decrease and subsequently AMPK is activated in cells. There are several upstream kinases that can activate AMPK by phosphorylating a threonine residue on its catalytic *α* subunit, liver kinase B1, calcium/calmodulin kinase and TGF-*β*-activated kinase-1 [35]. AMPK can activate autophagy via two independent mechanisms: suppression of mTORC1 activity and direct control of ULK1 phosphorylation [37].

mTOR is an evolutionarily conserved protein kinase and forms two functional complexes, termed mTORC1 and mTOR complex 2 [32, 33]. mTORC1 is a rapamycin-sensitive protein kinase complex and regulates a wide array of cellular processes including cell growth, proliferation, and autophagy in response to nutrients such as amino acids and growth factors [33, 38, 39]. mTORC1 activity reflects cellular nutritional status. Therefore, a better understanding of how mTORC1 regulates autophagy is of great importance because it may link nutrient signals to the regulation of autophagy. mTORC1 activity is finally and positively regulated by a lysosomal, membrane-anchored, small GTPase named Rheb [38–40].

Insulin signal phosphorylates protein kinase B (Akt) via PI3K and phosphoinositide-dependent kinase-1. Phosphorylated Akt suppresses tuberous sclerosis 2, a strong Rheb suppressor [38–40]. Therefore, an insulin signal suppresses autophagy via mTORC1 activation in cells through signal transduction.

Amino acids are also required for full activation of mTORC1 [41]; however, the mechanism of mTORC1 activation by amino acids is different from that of insulin. Recent research suggests that activation of mTORC1 by amino acids correlates with the translocation of mTORC1 from the cytoplasm to lysosomal membranes via Ras-related GTP-binding protein (Rag)-dependent system [42]. Activated mTORC1 can phosphorylate and inhibit Ulk1, which is a critical molecule in initiating autophagosome formation, leading to inhibition of autophagosome formation [20].

In addition to nutrient starvation, several intracellular stresses can induce autophagy. Reactive oxygen species (ROS) are small and highly reactive molecules that can oxidize proteins, lipids, and DNA. It has been reported that ROS induces autophagy through multiple mechanisms. Some reports have shown that exogenous hydrogen peroxide can activate PKR-like kinase (PERK), which subsequently phosphorylates eIF2a, oxidizes and activates Atg4 proteases [43], and thereby accelerates the production of proteolytic mature LC3 and inhibits mTORC1 activity. The cellular response to an increase in ROS often involves the activation of mitogen-activated protein kinases, including JNK1, which can activate autophagy [44, 45]. Furthermore, cells must remove damaged mitochondria to prevent the accumulation of ROS. This process of mitochondrial quality control is mediated by mitophagy, the selective autophagic removal of mitochondria. In response to potentially lethal stress or damage, mitochondrial membranes can be permeabilized through multiple distinct biochemical routes. The autophagic recognition of depolarized mitochondria is mediated by a refined voltage sensor, involving mitochondrial kinase, PINK1 accumulation [46, 47].

Hypoxia also activates autophagy. In response to hypoxia, HIF1 transcription factor is activated [48, 49] and induces the transcription of BNIP3 and NIX. Their protein products compete with Beclin-1 for the binding of BCL2, thereby releasing Beclin-1 and allowing it to induce autophagy [50].

Autophagy also plays an important role in the maintenance of the structural and functional integrity of the ER. ER is not only involved in protein synthesis and maturation but may also constitute a major source/scaffold of the autophagic isolation membrane [51]. The unfolded protein response (UPR), the major ER stress pathway [52], is a potent stimulus of autophagy. Three sensors located on the membrane of the ER are responsible for monitoring ER stress and initiating the UPR: inositol requiring ER-to-nucleus signal kinase-1, PERK, and activating transcription factor-6 (ATF6). Among these, PERK and ATF6 act as autophagy inducers [53]. PERK mediates the transcriptional activation of proteins LC3 and Atg5 through the action of transcription factors ATF4 and CHOP, respectively [54]. PERK may also reduce translation of I*κ*Ba, thereby activating NF-*κ*B, which also could contribute to autophagy. These intracellular stresses have recently been studied as a pathogenesis of diabetic nephropathy, in addition to the classical pathogenesis of diabetic nephropathy.

## 4. Autophagy in Podocytes under Diabetic Conditions

Podocytes are highly specialized, terminally differentiated, and unable to proliferate. Podocyte loss due to apoptosis and podocyte dysfunction contributes to proteinuria in patients with diabetic nephropathy [55–57]. Thus, maintaining podocyte cell homeostasis is regarded as a therapeutic target in diabetic nephropathy.

Autophagy is likely to play an essential role in maintaining podocyte function. Podocytes show high rates of autophagy even under nonstress conditions, suggesting that podocytes need to maintain cellular homeostasis by autophagy under basal conditions [9, 58–60]. In contrast, proximal tubular cells can proliferate and show low rates of autophagy under basal conditions [11, 14].

**Figure 3:**
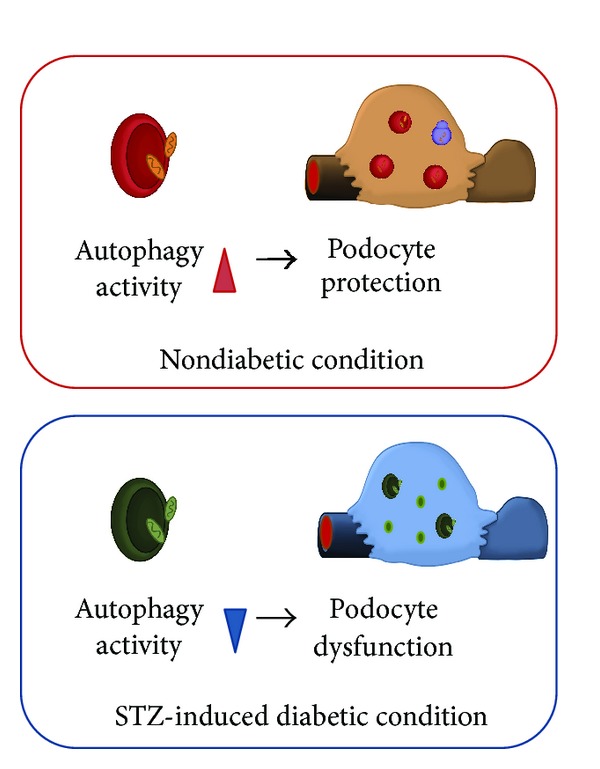
Autophagy in podocytes under diabetic conditions. Podocytes show high rates of autophagy even under nonstress conditions (upper panel). Autophagy activity is altered in podocytes under streptozotocin-induced type 1 diabetic conditions (lower panel). These results suggest that hyperglycemia alters autophagic activity, which may contribute to diabetes-related podocyte injury.

It has recently been reported that podocyte-specific autophagy depletion (*Atg5* gene depletion) leads to glomerulopathy in aging mice, accompanied by accumulation of oxidized and ubiquitinated proteins, ER stress, and proteinuria [9]. The role of autophagy in podocytes under diabetic conditions is still unclear. However, some reports suggest that autophagy may be involved in the pathogenesis of diabetic nephropathy. High-glucose conditions in cultured podocytes inhibit high basal autophagy by suppressing the expression of Beclin-1, Atg12-5, and LC3, and inhibition of basal autophagy impairs the filtration barrier function of podocytes [61]. Furthermore, this study reported that autophagy activity decreased in podocytes under streptozotocin (STZ)-induced type 1 diabetic conditions [61]. These results suggest that hyperglycemia reduces autophagic activity in podocytes, which may contribute to diabetes-related podocyte injury (Figure 3).

Autophagy activity is tightly associated with mTORC1 activity [62]. Interestingly, in podocytes of diabetic mice and patients, mTORC1 is highly activated and may be involved in the mechanisms of diabetes-related autophagy inhibition in podocytes [63]. Furthermore, podocyte hypertrophy is a predictor of renal lesion progression in patients with diabetes [64], and mTORC1 hyperactivation in the presence of hyperglycemia probably mediates a sustained hypertrophic stimulus that results in podocyte degeneration, the development of glomerulosclerosis and proteinuria [65]. These results suggest that the mTORC1-autophagy axis may be a future therapeutic target in diabetic nephropathy.

Rapamycin, a potent mTORC1 inhibitor, can ameliorate glomerular lesions in diabetic animal models [66, 67]. However, it is still not clear whether autophagy activation is involved in the mechanism underlying the rapamycin-mediated renoprotective effects in diabetes.

Some studies have reported that AMPK activation reduced podocyte permeability to albumin and podocyte dysfunction in STZ-induced diabetic mice [68]. In addition, several studies have reported that AMPK activation by AICAR or adiponectin shows podocyte protective effects against various nephrotoxic conditions. Although further evidence is required, it appears that autophagy activation is involved in AMPK-mediated podocyte protection.

## 5. Autophagy in Proximal Tubular Cells in Diabetic Nephropathy

The renal prognosis of diabetic patients with proteinuria is very poor compared with that of nondiabetic patients with proteinuria. Because proteinuria induces tubulointerstitial damage leading to progressive renal function decline, the diabetic condition may exacerbate proteinuria-induced proximal tubular cell damage leading to a poor renal outcome in diabetic patients with persistent proteinuria [1–3, 69]. Thus, identifying the mechanisms underlying the vulnerability of proximal tubular cells may lead to new therapies in diabetic patients.

The roles of autophagy in podocytes and proximal tubules are likely to be different. Autophagy activity in proximal tubular cells under basal conditions is very low compared with that in podocytes. Conversely, autophagy is extremely active under several nephrotoxic stresses, such as anticancer drugs and ischemia-reperfusion [11–15, 70]. Previous studies using proximal tubular cell-specific autophagy-depleted mice suggest that autophagy shows a renoprotective effect against acute kidney injury [11, 13, 14, 70]. It has also been clarified that the renoprotective effect of autophagy is not only against acute kidney injury but also chronic kidney damage, such as that with aging.

Proteinuria filtered from glomeruli has a nephrotoxic effect in proteinuric kidney diseases including diabetic nephropathy [2, 3, 69]. In an experimental mouse model that induced proteinuria-induced tubulointerstitial lesions, autophagy was activated, especially in proximal tubular cells that reabsorbed proteinuria. Of note, proteinuria-induced tubular cell damage was exacerbated in the kidneys of proximal tubular cell-specific autophagy-depleted mice, and obesity significantly suppressed proteinuria-induced autophagy. Obesity-mediated autophagy deficiency is therefore likely to be involved in the pathogenesis of the vulnerability of proximal tubular cells under diabetic conditions [71]. It appears that autophagy has a renoprotective role in proximal tubular cells under both acute and chronic conditions.

**Figure 4:**
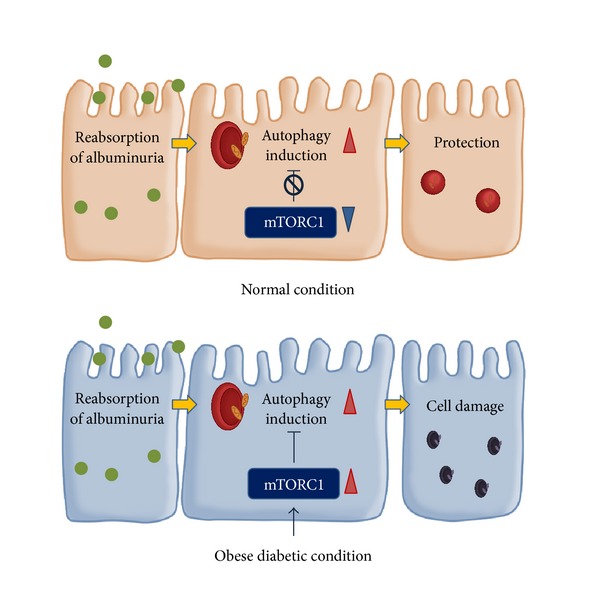
Autophagy in proximal tubular cells under diabetic conditions.   Proximal tubular cells show low rates of autophagy under basal conditions. Proteinuria renoprotectively elicits autophagy in proximal tubular cells (upper panel). Obesity suppresses proteinuria-induced autophagy via hyperactivation of mTORC1 in proximal tubular cells, leading to obesity-mediated exacerbation of proteinuria-induced tubulointerstitial damage (lower panel).

A recent study by the authors suggests that obesity significantly suppressed the renoprotective action of autophagy in proximal tubular cells, and autophagy insufficiency was confirmed in renal biopsy specimens from patients with type 2 diabetes or obesity with proteinuria [71]. In that study, we also examined the mechanisms underlying autophagy deficiency-induced proximal tubular cell damage in high-fat diet-induced obese mice and patients with type 2 diabetes or obesity with proteinuria [71]. The results suggested that hyperactivation of mTORC1 signaling in proximal tubular cells was involved in obesity-mediated autophagy suppression (Figure 4) [71]. Interestingly, obesity-mediated suppression of proteinuria-induced autophagy was recovered by diet restriction and treatment with rapamycin, a specific inhibitor of mTORC1 signaling, suggesting that obesity-mediated autophagy deficiency is a reversible phenomenon [71]. A recent study has reported that dietary restriction ameliorates diabetic nephropathy through anti-inflammatory effects and regulation of autophagy via restoration of sirt1 in diabetic Wistar fatty (fa/fa) rats [72]. Therefore, restoration of autophagy activity may be a new therapeutic target for overt proteinuria in patients with diabetic nephropathy.

What kinds of metabolic alterations are associated with diabetes- and obesity-related autophagy insufficiency in proximal tubular cells? In diabetic conditions, hyperglycemia, hyperinsulinemia, and higher level of plasma free fatty acids are major metabolic alterations caused by the insufficient insulin actions to insulin-sensitive organs, hepatocytes, skeletal muscle cells, and adipocytes. Based on our recent study, free fatty acids caused autophagy in response to lipotoxicity-related intracellular stress [71]. It has been well known that glucose and insulin are able to inhibit autophagy in various cells. Thus, hyperglycemia and hyperinsulinemia rather than higher level of plasma fatty acids may contribute to diabetes-related suppression of autophagy in the kidney. Further examinations are needed to conclude it.

## 6. Drug Discovery Targeting Autophagy

Drug discovery research aimed to regulate autophagy is in progress worldwide. There are some chemical mediators that stimulate macroautophagy [73, 74]; however, no drug has yet been developed that stimulates microautophagy or chaperone-mediated autophagy. Drug research is currently focused on regulating autophagy in several degenerative and malignant diseases.

As mentioned above, autophagy activity is suppressed under diabetic conditions. We have therefore focused on a strategy for the resumption or activation of autophagy. There are two major strategies for activation of autophagy: the mTORC1-dependent pathway and the mTORC1-independent pathway.

Rapamycin is a potent activator of autophagy via the inhibition of mTORC1 [75] and appears to ameliorate mesangial expansion, glomerular basement membrane thickening, and renal macrophage recruitment in type 1 diabetic rats and to prevent proteinuria [67]. Thus, an mTOR inhibitor, such as rapamycin, has been the focus as a type of drug for that treatment of diabetic nephropathy via autophagy activation. However, other reports have suggested that complete inhibition of mTORC1 signaling by treatment with rapamycin exacerbates glomerular damage along with proteinuria in animal and human studies [76, 77]. This is a major adverse effect of mTORC1 inhibitor. Long-term mTOR inhibition may be associated with induction of malignancy as well as proteinuria, because it is well established that the mTOR pathway is necessary for activating the immune system [78]. Furthermore, recent studies suggest that mTOR inhibitor induces insulin resistance. In this respect, nonspecific mTORC1 inhibition, including rapamycin treatment, is harmful although it can activate autophagy. A mechanism specific to mTORC1-dependent autophagy suppression needs to be identified to develop safer drugs to activate autophagy via mTORC1 suppression.

As described above, AMPK activation is also a potent activator of autophagy. It has been revealed that AMPK stimulators such as metformin can work as autophagy activators [79]. There are few reports detailing its adverse effects on human and animal health [80, 81]. If pharmacological AMPK activation really acts as an autophagy activator, a drug that stimulates AMPK may be a potential therapy for diabetic nephropathy [82]. Several studies have reported that AMPK activation shows renoprotective effects in diabetic nephropathy. Autophagy may be involved in AMPK-mediated renoprotective action.

Previous reports suggest that trehalose, a disaccharide, activates autophagy in a mTORC1-independent manner [83]. Several antiepileptic and mood stabilizer drugs (e.g., LiCl, valproate, and carbamazepine) also have the ability to activate autophagy and degrade proteins via the mTORC1-independent pathway in some types of cells [84, 85]. Unfortunately, the mechanisms underlying autophagy activation with these drugs remain unknown, and it is therefore inappropriate to prescribe these drugs to patients with diabetes. Discovery for an autophagy regulator is just the beginning.

## 7. Discussion and Conclusion

Although the body of evidence is still small, relative autophagy insufficiency may be involved in the exacerbation of diabetic nephropathy, and several medications have the potential to activate autophagy. The authors therefore expect that autophagy activation will become a potential therapy to combat diabetic nephropathy. However, at present, there are a couple of major problems to be resolved as described below.

Further examinations are needed to conclude whether autophagy activation is a safe therapy for any kidney diseases, since some reports have suggested that autophagy activation was associated with tubular cell damages in some acute kidney injury models [86–88].

The role of autophagy in the development of diabetes is still under debate. Some investigators have shown that pancreatic *β*-cell-specific autophagy-deficient mouse developed glucose intolerance [89, 90], whereas others have reported that autophagy activation led to *β*-cell apoptosis [91]. Furthermore, recent interesting studies have shown that genetic inhibition of autophagy in adipose tissue, skeletal muscle, and liver prevented the development of high-fat diet-induced obesity [92–94]. Therefore, it remains unclear whether autophagy activation shows a health beneficial effect in all stages of diabetic diseases, from the onset of diabetes mellitus to the progression of diabetic complications.

In the past decade, several genetic links have emerged between autophagy deficiency and cancer development, providing increasing support for the concept that autophagy is a tumor suppressor pathway [95]. In contrast, several studies have shown that genetic or pharmacological inhibition of autophagy enhances cytotoxicity of cancer chemotherapeutic agents [96]. The incidence rate of malignant disorder is higher in patients with diabetes. Thus, further examinations regarding the pathogenic and therapeutic roles of autophagy in cancer biology are required.

We have no technical tool to detect autophagy activity in the kidney of humans; therefore the discovery of a biomarker for autophagy activity with serum and/or urine samples is urgently needed. Finally, no drug has been discovered that can activate autophagy without adverse effects.

When the abovementioned problems are overcome, autophagy regulation may be an effective therapeutic target for diabetic nephropathy. We hope this review has been helpful to researchers interested in autophagy and diabetic nephropathy.
